# Critical role of C-terminal residues of the Alzheimer's associated β-amyloid protein in mediating antiviral activity and modulating viral and bacterial interactions with neutrophils

**DOI:** 10.1371/journal.pone.0194001

**Published:** 2018-03-16

**Authors:** Mitchell R. White, Ruth Kandel, I-Ni Hsieh, Xavier De Luna, Kevan L. Hartshorn

**Affiliations:** Boston University School of Medicine, Department of Medicine, Boston, MA, United States of America; Hospital for Sick Children, CANADA

## Abstract

Recent studies have shown that the Alzheimer’s associated β-amyloid protein (βA) can inhibit growth of bacteria, fungi and viruses. We reported that the 42 amino acid βA protein inhibits replication of seasonal and pandemic strains of H3N2 and H1N1 influenza A virus (IAV) in vitro and modulates activation of neutrophils and monocytes exposed IAV. We here show that fragments composed of the N and C terminal domain of βA42, including βA22-42 and the 8 amino acid βA35-42, retain viral neutralizing and viral aggregating activity, whereas fragments lacking the C-terminal amino acids 41 and 42 (e.g. βA1-40, βA1-34, βA1-28, βA22-40 or βA33-40) have markedly diminished activities on these assays. βA22-42 also increased viral uptake, and virus induced respiratory burst responses, by human neutrophils, while peptides lacking residues 41 and 42 did not. Similar results were obtained with regard to bacterial aggregation, or promotion of bacterial uptake by neutrophils. Published structural studies have shown that βA1-42 has a greater propensity to form neurotoxic oligomers than βA1-40 due to a molecular interaction between Met35 and Ala42. Our findings suggest that there is a relationship between neurotoxic and antimicrobial activities of βA1-42. Truncated peptides containing the last 8 C-terminal amino acids of βA1-42 retain antimicrobial and opsonizing activities likely resulting from their ability to induce viral or bacterial aggregation.

## Introduction

Accumulation of the Alzheimer’s associated β-amyloid protein (βA) is believed to contribute strongly to the pathogenesis of Alzheimer’s disease (AD), although the actual physiological function and reason for accumulation of βA in the brain are not known. βA peptides are fragments of the larger β amyloid transmembrane precursor protein (APP. βA1-40 is more abundant than βA1-42, but xA1-42 is the more amyloidogenic and neurotoxic species [1–3]. The neurotoxicity of βA1-42 has been shown to depend on the ability of this peptide to form unstable oligomers (pentamers mainly), whereas the protofibrils or fibrils formed from the peptide are less neurotoxic [4].

There are a variety of studies suggesting links between βA or AD and inflammation or infection [5]. Excess accumulation of βA has been linked to Alzheimer’s disease but also to Human Immunodeficiency virus (HIV) related dementia [6], Herpes Simplex Virus (HSV) induced encephalitis [7], and cytomegalovirus infection. Examination of AD brain tissue has shown evidence of fungal infection. These findings suggest that viruses that infect the brain could be triggers for accumulation of βA, perhaps as part of an aberrant or sustained innate immune response.

The structure of βA resembles that of antimicrobial peptides like porcine protegrin and, like protegrin, it can form membrane channels [8]. Importantly, recent studies have demonstrated antibacterial and antifungal activity of βA peptides [9, 10]. We showed that βA1-42 inhibited influenza A virus (IAV) through a mechanism that involves viral aggregation. βA1-40 had significantly reduced antiviral activity in these studies as compared to βA1-42. βA has also since shown to have antiviral activity against HSV [11]. Recently Kumar et al showed that transgenic expression of βA1-42 protects against bacterial and fungal infection in mice and worms through a mechanism that involves agglutination of these microbes [12]. They also demonstrated that mice lacking βA due to gene knockout had increased mortality from bacterial meningitis. Most recently, Spitzer et al showed that bacterial and fungal aggregating activities of βA1-42 are retained in peptides βA2-42 or 3–42, but lost in βA1-40, again suggesting that the final two amino acids of βA1-42 may be important to antimicrobial activity [13]. Overall these studies indicate that βA peptides have broad spectrum activity against various potential pathogens (viral, bacterial and fungal), perhaps providing the long sought physiological role for βA.

Antimicrobial peptides can also trigger recruitment and activation of immune cells [14]. βA peptides have been shown to activate glial cells and macrophages [15] in part through binding to toll-like receptor 2 (TLR2) as well as other receptors [16]. We demonstrated that βA1-42 modulates responses of neutrophils and monocytes to IAV [17]. βA1-42 increased neutrophil uptake of IAV and potentiated neutrophil respiratory burst and extracellular trap (NET) formation in response to the virus [17]. Of note, βA1-40 lacked the ability to either increase neutrophil uptake or neutrophil respiratory burst responses to IAV. These findings suggest that βA1-42 can modulate phagocyte responses to pathogens, and that this property also depends on the C-terminal amino acids Ile41 and Ala42.

In this paper we extend on our prior findings to determine the key antiviral domains of βA1-42. Through study of additional peptide fragments of βA1-42 we confirm the importance of Ile41 and Ala42 in mediating antiviral, bacterial aggregating, and neutrophil modulating effects of the protein. In addition we show that significantly shortened peptides containing these amino acids retain or exceed antiviral and/or neutrophil activating activities of βA1-42.

## Methods

### Ethics statement

Blood collection for isolation of neutrophils and monocytes was done with informed consent as approved by the Institutional Review Board of Boston University School of Medicine. The Institutional Review Board specifically approved this study and also approved the consent form for the study. The blood donors were healthy volunteers and they all signed the written consent form prior to each donation.

### Virus preparation

Philippines 82/H3N2 (Phil82) strain was kindly provided by Dr. E. Margot Anders (Univ. of Melbourne, Melbourne, Australia) and grown in the chorioallantoic fluid of ten day old chicken eggs and purified on a discontinuous sucrose gradient as previously described [18]. The virus was dialyzed against PBS to remove sucrose, aliquoted and stored at -80°C until needed. Post thawing the viral stocks contained ~5x10^8^ infectious focus forming units/ml. Rhodamine labeled *Escherichia coli (E*.*coli)* and *Staphylococcus aureus (S*.*aureus)* were purchased from Invitrogen (Carlsbad, CA).

### βA preparations

βA1-42, 1–40, 1–34, 1–28, 22–40, 22–42, 33–40, and 35–42 peptides were obtained from Genscript, Piscataway, NJ. These samples were tested for LPS and amounts were not detectable (i.e. <0.016 EU/ml) in βA peptides βA1-40, 35–42 and 33–40. Low levels of endotoxin were detected in βA1-42, 22–42, 22–40, 1–28 and 1–34 (respectively, 0.21, 2.2, 0.004, 0.74, and 0.27 EU per ml). The endotoxin assays were done using the highest concentrations of βA peptides used in the assays in the paper (50μg/ml). The highest concentration found was 2.2 EU/ml in βA22-42 which ≈1.4 pg/ml of endotoxin. Concentrations considerably higher than this did not have any effect on neutralization, aggregation or neutrophil functional assays in control experiments using purified endotoxin (n≥4 experiments for each assays). Table 1 shows the peptides used in this paper.

**Table 1:** Peptide sequences of βA derived peptides used in this paper.

Peptide	Amino Acid Sequence
**βA1-42**	DAEFRHDSGYEVHHQKLVFFAEDVGSNKGAIIGLMVGGVVIA
**βA1-40**	DAEFRHDSGYEVHHQKLVFFAEDVGSNKGAIIGLMVGGVV
**βA1-34**	DAEFRHDSGYEVHHQKLVFFAEDVGSNKGAIIGL
**βA1-28**	DAEFRHDSGYEVHHQKLVFFAEDVGSNK
**βA22-42**	EDVGSNKGAIIGLMVGGVVIA
**βA22-40**	EDVGSNKGAIIGLMVGGVV
**βA35-42**	MVGGVVIA
**βA33-40**	GLMVGGVV

### Fluorescent focus assay of IAV infectivity

MDCK cell monolayers were prepared in 96 well plates and grown to confluence. These layers were then infected with diluted IAV preparations for 45 min. at 37°C in PBS. MDCK cells were tested for presence of IAV infected cells after 18 hours of virus addition using a monoclonal antibody directed against the influenza A viral nucleoprotein (Millipore, Billerica, Ma) as previously described. IAV was pre-incubated for 30 min. at 37°C with various concentrations of βA or control buffer, followed by addition of these viral samples to the MDCK cells.

### Measurement of viral and bacterial aggregation by βA peptides

Viral aggregation caused by βA was measured by assessing light absorbance at 350nM by suspensions of IAV. This was done using a Perkin Elmer Lambda 35 UV/Vis spectrophotometer as described [19]. Viral aggregation was also assessed by electron microscopy (EM) as previously described [17]. Rhodamine labeled *E*.*coli* was incubated with βA preparations and then examined by fluorescent microscopy [20].

### Confocal microscopy

For these experiments the IAV was labeled with Alexa Fluor 594. Alexa Fluor 594 carboxylic acid, succinimidyl ester labeling kit was purchased from Molecular Probes and labeling was carried out using manufacturer’s recommendations with some modifications. In brief, concentrated virus stock was incubated with the Alexa Fluor in sodium bicarbonate buffer (pH 8.3) for one hour at room temperature. The preparation was then dialyzed overnight against PBS at 4°C. After this procedure there was no reduction in viral hemagglutination titer. MDCK cells were pre-incubated with the labeled virus for 45 min., followed by washing and fixation using 1% paraformaldehyde. Prior to this the IAV was either pre-incubated with control buffer or βA for 30 minutes at 37°C in the same manner as in the infectious focus assay. Wheat germ agglutinin (WGA)-Oregon Green 488 (4μg/ml) and DAPI 350 were used to stain the cell membrane and nucleus respectively. Confocal pictures were taken at Zeiss LSM510 (LSEB) on 100x resolution.

### Human neutrophil and monocyte preparation

Neutrophils from healthy volunteers were isolated to > 95% purity by using dextran precipitation, followed by Ficoll-Paque gradient separation for the separation of mononuclear cells (layering above the Ficoll-Paque) and neutrophils (below the Ficoll-Paque). The neutrophils were purified further by hypotonic lysis to eliminate any contaminating erythrocytes, as previously described [21]. Cell viability was determined to be >98% by trypan blue staining. The isolated neutrophils were re-suspended at the appropriate concentrations in control buffer (PBS) and used within 2 hours.

### Measurement of IAV and bacterial uptake by neutrophils

Fluorescein isothiocyanate (FITC)-labeled IAV (Phil82 strain) was prepared and uptake of virus by neutrophils was measured by flow cytometry as described [22]. In brief, IAV was treated with various doses of βA peptides for 30 min at 37°C. Then it was incubated with cells for 45 minutes at 37°C in presence of control buffer. Trypan blue (0.2 mg/ml) was added to these samples to quench extracellular fluorescence. Following washing, the neutrophils were fixed with 1% paraformaldehyde and neutrophil associated fluorescence was measured using flow cytometry. The mean cell fluorescence (>2000 cells counted per sample) was measured. Similar method was used for measuring neutrophil uptake of rhodamine labeled *E*.*coli* or *S*.*aureus*.

### Measurement of neutrophil H_2_O_2_ production

H_2_O_2_ production was measured by assessing reduction in scopoletin fluorescence as previously described [23]. Measurements were made using a POLARstar OPTIMA fluorescent plate reader (BMG Labtech, Durham NC).

### Statistics

Statistical comparisons were made using Student's paired, two-tailed *t* test or ANOVA with post hoc test (Tukey’s). ANOVA was used for multiple comparisons to a single control. Statistics were performed using Excel and Statmost programs.

## Results

### Neutralization of IAV strains by βA peptides

**Fig 1:**
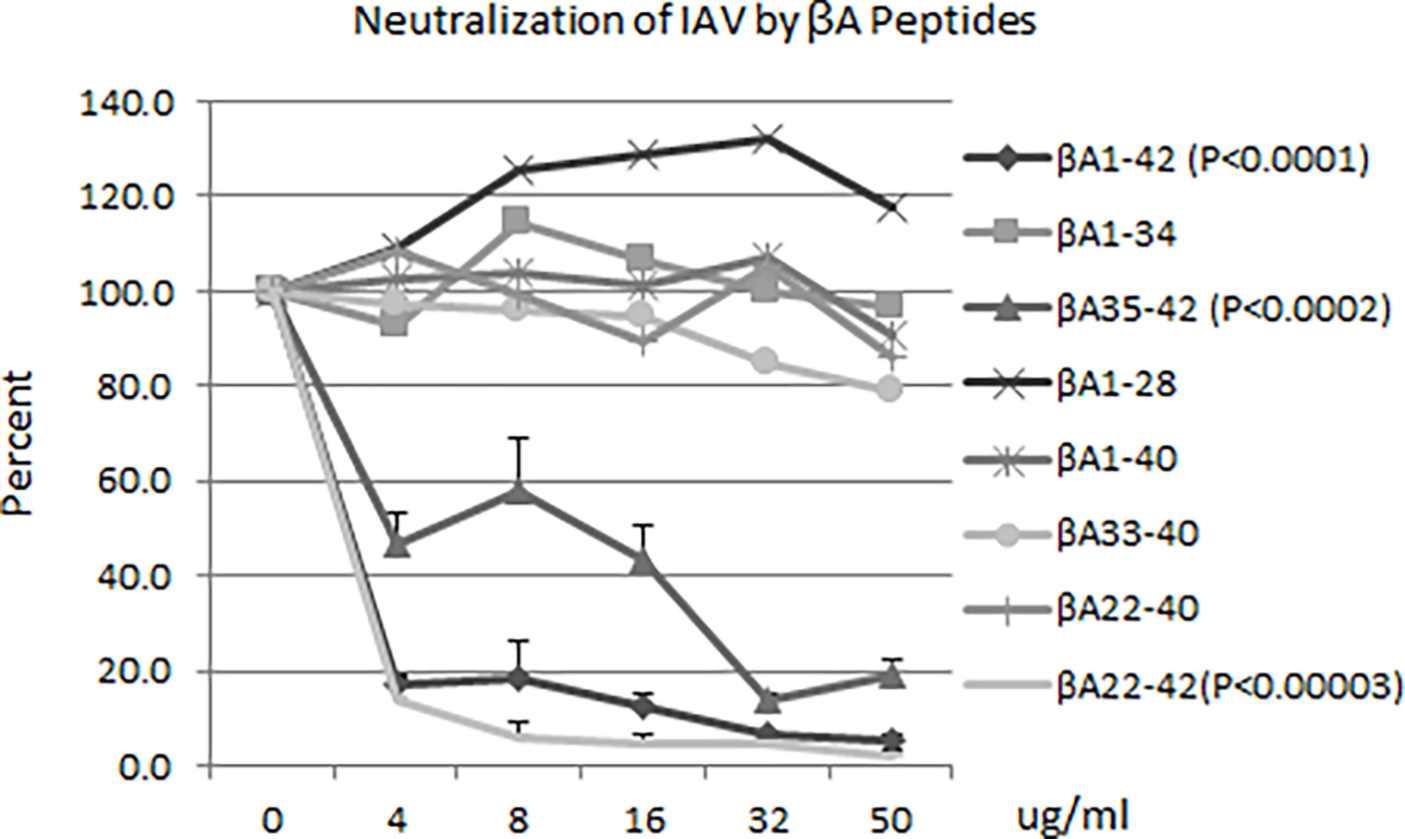
IAV neutralization by segments of beta-amyloid. Peptides in PBS were pre-incubated with Phil IAV H3N2, then used to infect confluent MDCK cells. The cells were fixed after 18 hours and the replicating viral NP protein were fluorescently labeled. Positive cells were counted on a fluorescent microscope and compared to Phil IAV alone. Peptides that contain Ile 41 and Ala42 retain neutralizing activity as did residues that contain the salt bridge between Lys28 and Asp23. P-values for BA1-42, BA35-42, and BA22-42 are shown. Results represent mean±SEM of 5 experiments.

As shown in Fig 1, several βA peptides significantly inhibited infectivity of a seasonal H3N2 strain of IAV (Phil82), including βA1-42, βA22-42, and βA35-42. βA1-40, βA1-34, βA1-28, βA22-40 and βA33-40, had no or minimal antiviral activity in these experiments. We previously showed that βA1-40 has lower inhibitory activity than βA1-42 for this strain [17]. These results suggest that the C-terminal amino acids Iso41 and Ala42 are critical for antiviral activity. It is notable that the 8 amino acid peptide βA35-42 retained antiviral activity. We tested all of the peptides to determine if they altered cell viability using and LDH assay and no increase in LDH release was noted for any peptide at a 50μg/ml concentration (S1 Fig).

### Aggregation of IAV by βA peptides

**Fig 2:**
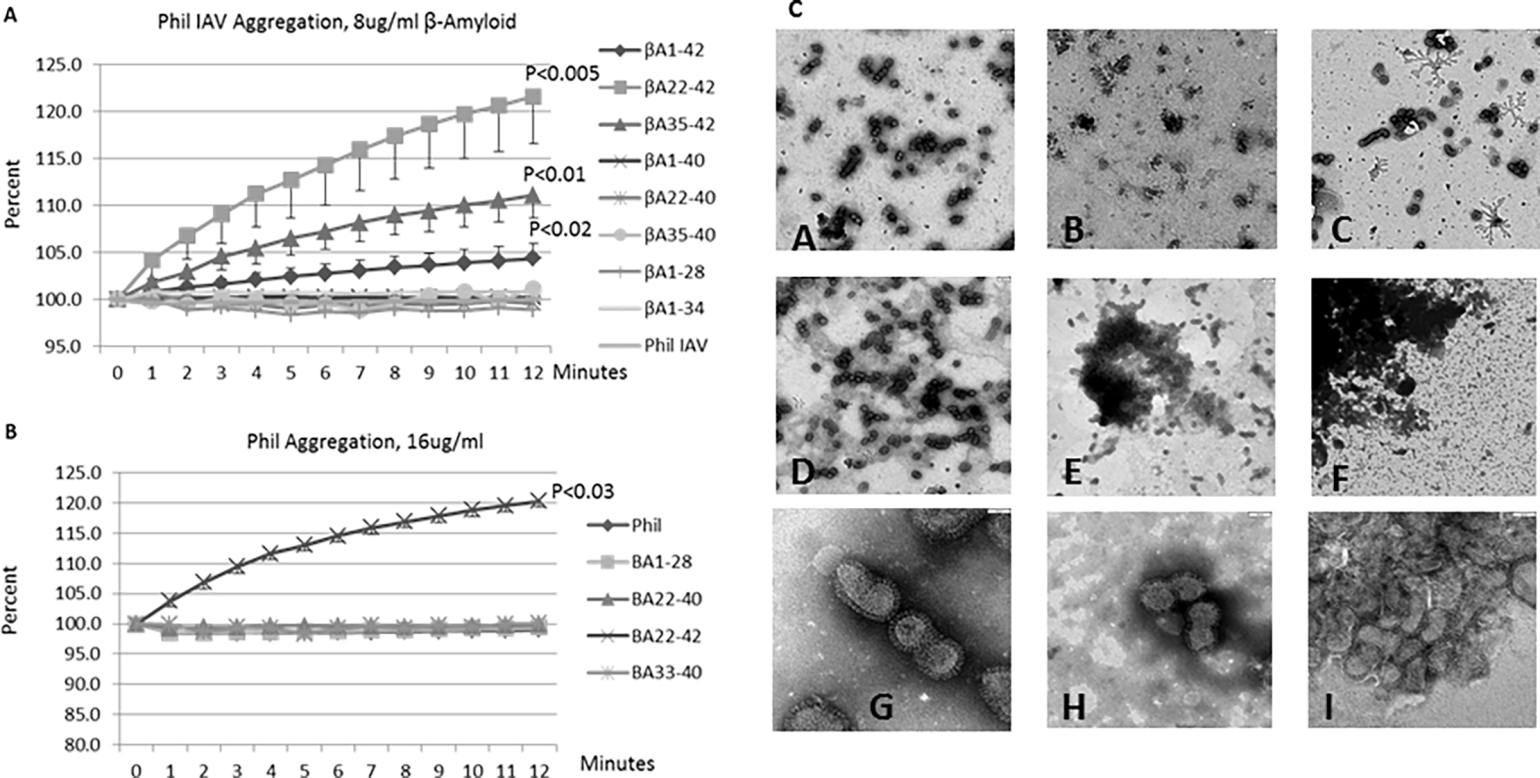
Viral aggregation induced by βA peptides. **A and B. Measurement of aggregation by light transmission.** The βA peptides were added to a dilute suspension of IAV and light transmission was monitored on a Lambda UV/Vis spectrometer at 350nm wavelength. Panel A shows results with 8μg/ml of βA peptides. The βA peptides containing Ile41 and Ala42 resulted in the most viral aggregation over 12 minutes. At 12 minutes p-values are for βA peptides as compared to control virus alone are shown. Panel B shows results with 16μg/ml of the βA peptides. Again the βA peptides lacking Ile41 and Ala42 caused no aggregation, whereas βA22-42 caused statistically significant aggregation as noted. Results obtained with a highly aggregating concentration of SP-D are shown for comparison. **C. Electron Microscopy.** Electron micrographs were taken at either 5,600x magnification (images A-F) or 40,000x magnification (images G-H). Images A and G show control virus, images B and C showed virus treated with 2 or 8μg/ml of βA22-40. Images E and F show virus treated with 2 or 8μg/ml of βA22-42. Image D shows virus treated with 10 EU/ml as an additional control. Images G, H and I show virus control, virus treated with 8μg/ml of βA22-40 and 2μg/ml of βA22-42, respectively. The images are representative of 3 similar experiments.

**Fig 3:**
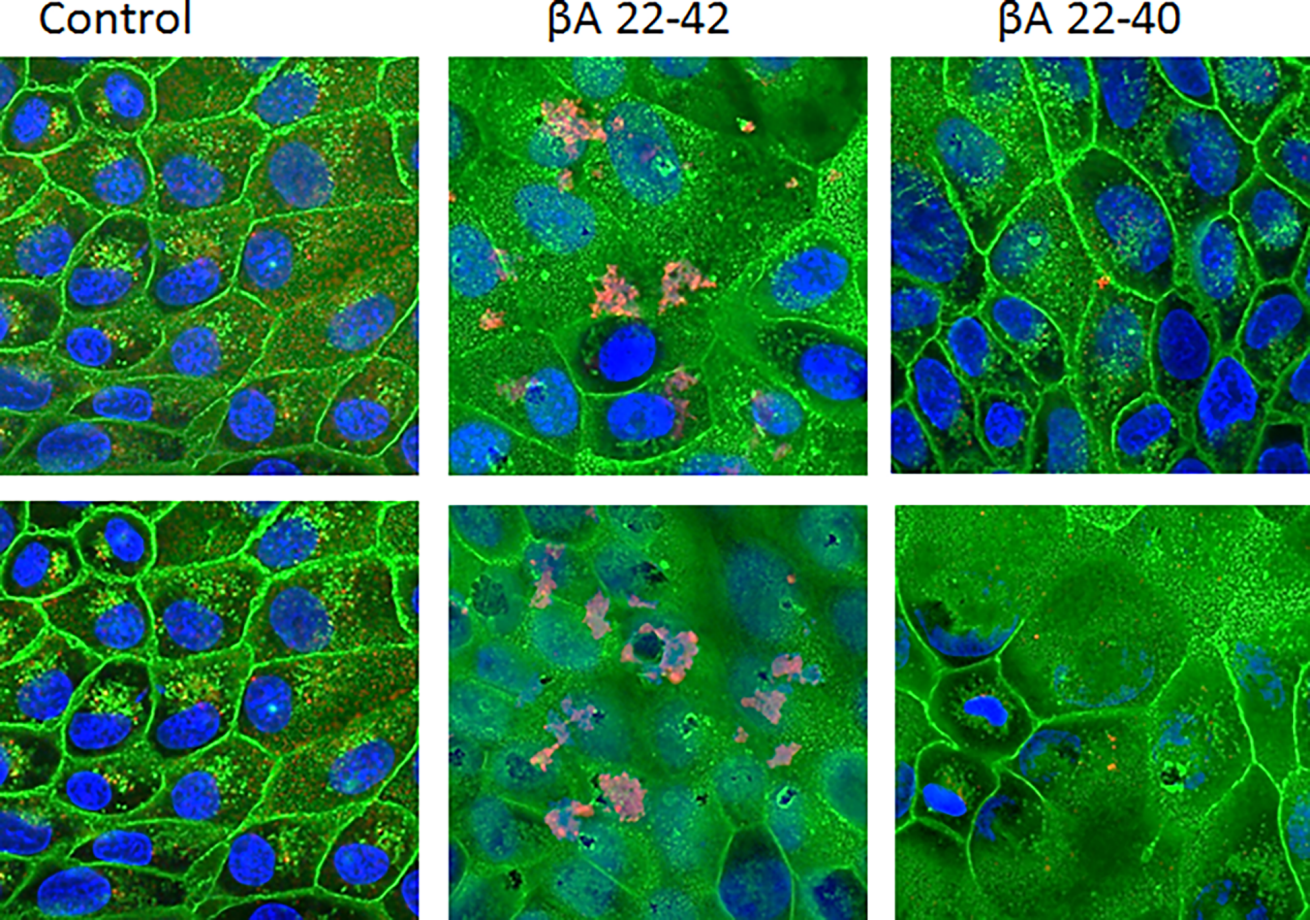
Confocal images of IAV incubated with MDCK cells—Alexafluor 594-labeled IAV was incubated with control buffer, βA22-40, or βA22-42 prior to addition to MDCK cell monolayers as in the infectious focus assay. The virus appears red, cell nuclei were stained with Dapi 350 and appear blue, and cell membranes were labeled with WGA-Oregon Green 488. Aggregates were apparent in samples treated with βA22-42 but not in samples treated with βA22-40. Results shown are from two separate experiments (total of 3 performed with similar results). The pictures were taken at 100x magnification.

As shown in Fig 2A and 2B, βA peptides 1–42, 35–42, and 22–42 also caused aggregation of IAV particles, whereas the remaining peptides did not. βA22-42 had the highest activity in this assay. By comparison, βA peptides that lacked the last two amino acids did not cause aggregation of IAV. We also evaluated viral aggregation using electron microscopy (EM) as shown in Fig 2C. Results obtained with surfactant protein D (SP-D) which has well established ability to aggregate IAV and bacteria are presented for comparison in Fig 2B. βA22-42 caused marked viral aggregation, whereas βA22-40 did not. LPS did not cause any viral aggregation (Fig 2C panel D). We also tested viral aggregation under the conditions of the fluorescent focus assay using confocal microscopy. Aggregates were visible in cases where Alexafluor labeled virus was pre-incubated with βA22-42 but not when pre-incubated with βA1-40 (Fig 3).

### βA peptides containing C-terminal amino acids increased neutrophil uptake of IAV

**Fig 4:**
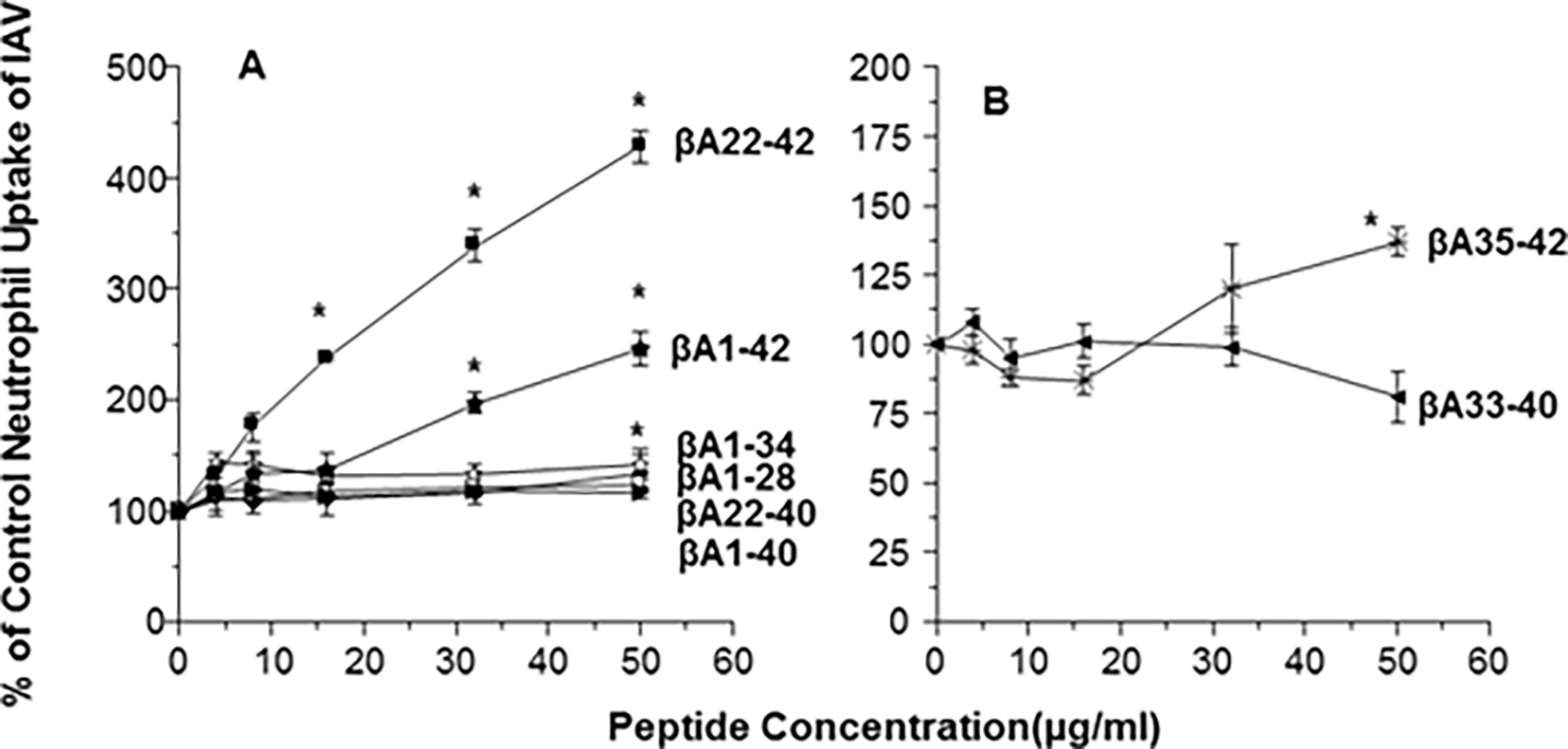
Neutrophil uptake of IAV after incubation with βA peptides. βA peptides at the indicated concentrations were pre-incubated with FITC-conjugated IAV followed by incubation with neutrophils as described. External fluorescence was quenched using trypan blue. Viral uptake was analyzed on a FacScan flow cytometer. Panel A shows results using βA1-42, βA1-34 and βA1-28, βA22-42 and βA22-40. Panel B shows results using βA35-42 and βA33-40. Results represent mean±SEM of 5 experiments using separate neutrophil donors. * indicates p<0.05 compared with control.

We previously showed that βA1-42 significantly increases neutrophil uptake of IAV. We tested activity of βA1-42 in parallel with the other peptides in this assay (Fig 4). βA1-42 and 22–42 had the ability to increase viral uptake by human neutrophils. For βA1-42 and βA22-42 the activity was dose dependent, with βA22-42 showing greater increases than βA1-42 (Fig 4A). βA1-40, 33–40, 22–40, 1–34 and 1–28 lacked activity in this assay (Fig 4A and 4B). βA35-42 increased uptake at the highest concentration tested (Fig 4B).

### Effect of βA peptides on neutrophil H_2_O_2_ responses to IAV

**Fig 5:**
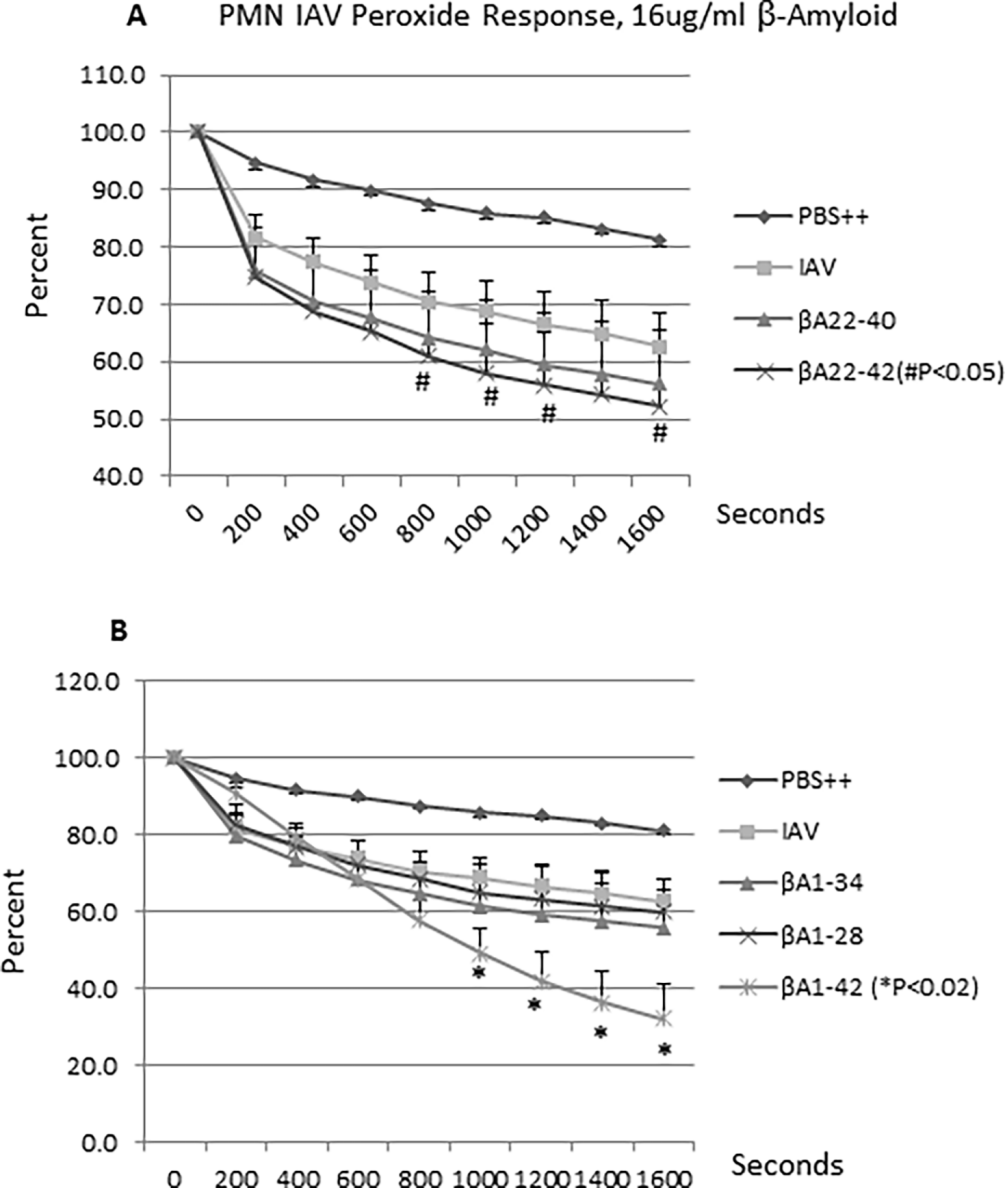
Effects of βA peptides on neutrophil respiratory burst responses to IAV. IAV was pre-incubated with βA peptides followed by addition to neutrophils and measurement of neutrophil H_2_O_2_ release as measured by decrease in scopoletin fluorescence. Panel A shows that βA22-42 caused increased H_2_O_2_ release as compared to IAV alone. Panel B compares results of βA1-42 with βA1-28 and βA1-34. Results represent mean±SEM of 5 experiments using separate neutrophil donors. p values for βA22-42 and βA1-42 compared to IAV alone are indicated.

H_2_O_2_ production by neutrophils was measured based on its ability to quench the fluorescence of scopoletin. Cells stimulated with IAV alone produced more H_2_O_2_ than cells in PBS buffer alone. Pre-incubation of IAV with 16μg/ml of βA22-42 caused increased H_2_O_2_ formation as compared to IAV alone and slightly increased production compared to IAV pre-incubated with βA22-40 (Fig 5A). βA 35–42 (not shown) and the β-amyloid peptides lacking the last two C-terminal amino acids did not increase H_2_O_2_ production (Fig 5B). For comparison βA1-42 was shown to significantly increase the H_2_O_2_ response as previously reported (Fig 5B).

### Effect of βA peptides on E.coli aggregation and uptake of E.coli by neutrophils

**Fig 6:**
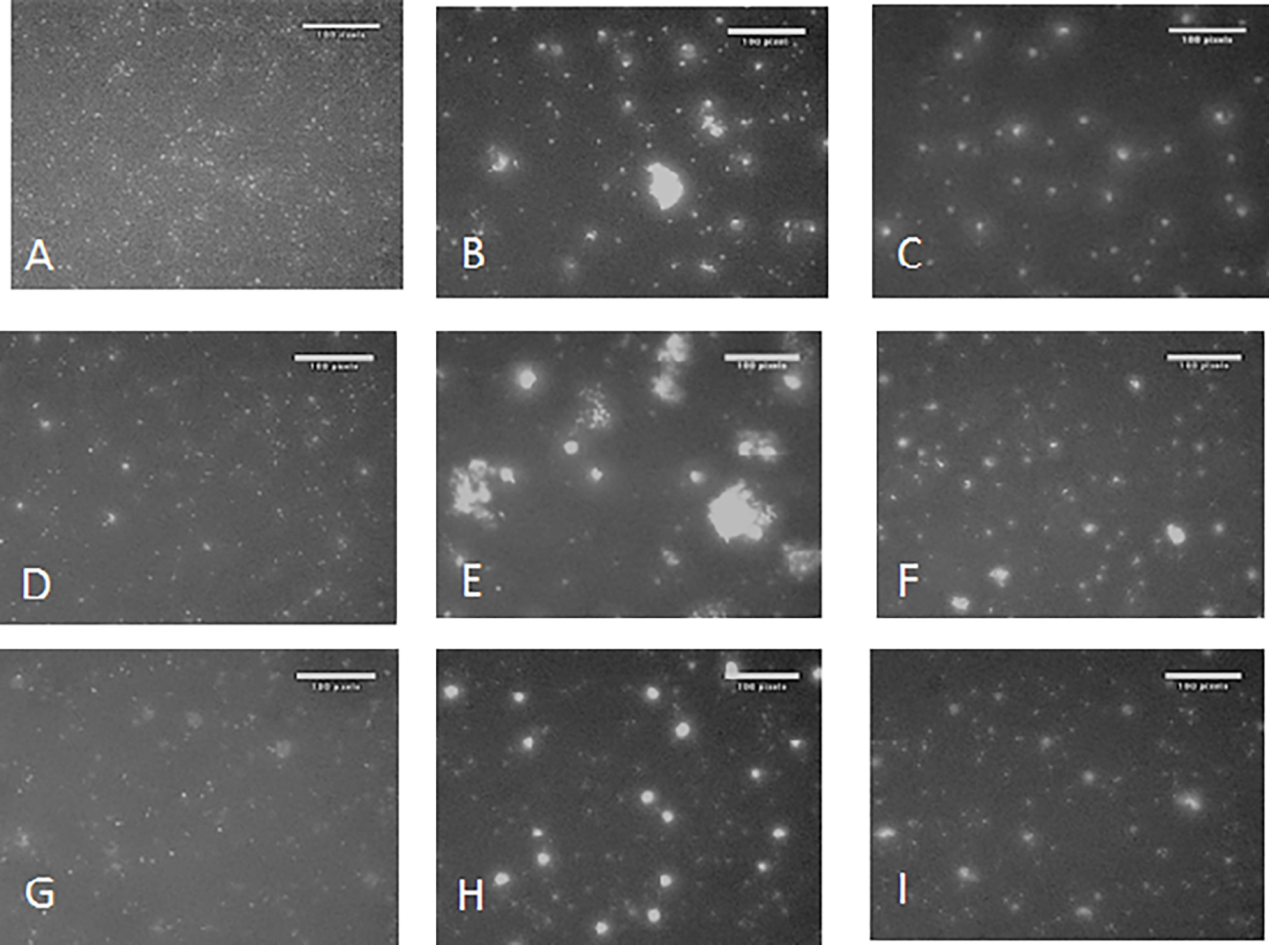
Aggregation of E.coli by βA peptides. Rhodamine conjugated E. coli preincubated with 50ug/ml of βA peptides for 1 hour at 37°C. Photographs were taken on a fluorescent microscope with a 20X objective. Panels A, D and G show samples treated with control buffer alone; panels B, E and H show samples treated with βA1-42, βA22-42, and βA35-42; panels C, F and I shows samples treated with βA1-40, βA22-40, and βA33-40. These are representative samples of three experiments all showing greater aggregation when the last two amino acids were present.

**Fig 7:**
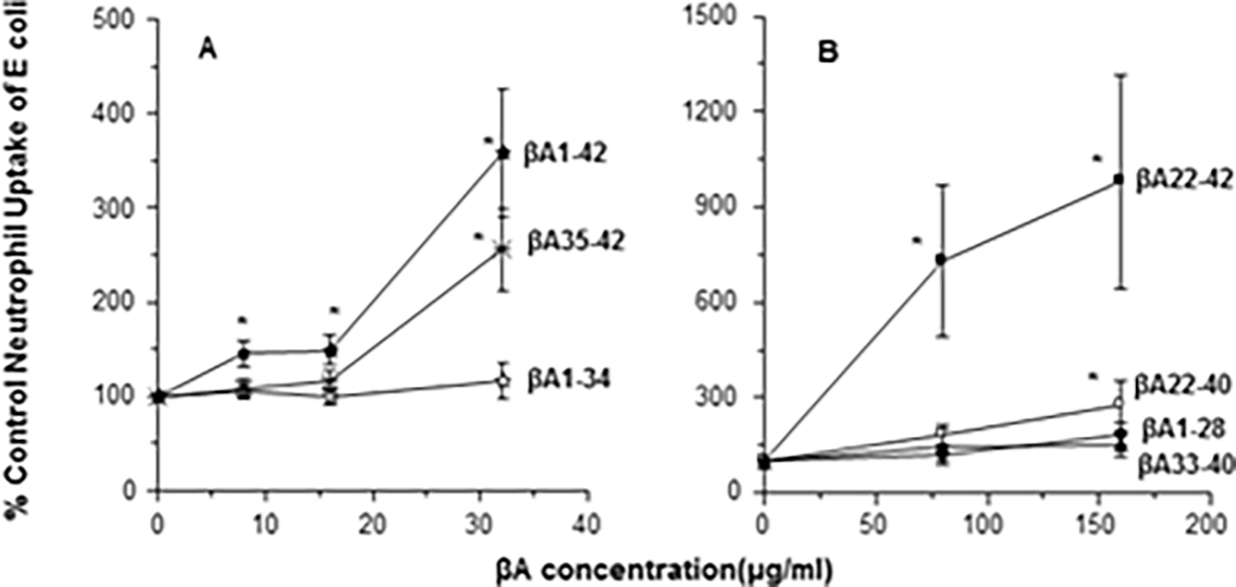
Neutrophil *E*. *coli* uptake alone or pre-incubated with the β-amyloids. Panel A shows the effects of pre-incubation of rhodamine labeled *E*.*coli* with βA1-42, βA35-42 or βA1-34 on subsequent neutrophil uptake of the bacteria. Both βA1-42 and βA35-42 increased neutrophil uptake of the bacteria, but βA1-34 did not. Panel B shows additional experiments in which the effects of βA22-42 were compared to those of βA22-40, βA1-28 and βA33-40. βA22-42 caused marked uptake of the bacteria as measured by neutrophil associated fluorescence. βA22-40 caused slight but significantly increased uptake at the highest concentration tested. The other peptides did not alter bacterial uptake. Results represent mean±SEM of 5 experiments using separate neutrophil donors. * indicates p<0.05 compared with control.

To confirm our findings using a bacteria instead of virus we tested the ability of the peptides to aggregate or increase neutrophil uptake of *E*.*coli*. As shown in Fig 6, βA1-42 and βA 22–42 caused aggregation of fluorescent *E*.*coli* but βA22-40 did not. We also tested whether βA peptides could increase uptake of bacteria by neutrophils. As shown in Fig 7A, βA1-42 caused significant increase in uptake of fluorescently labeled *E*.*coli*. βA1-42 also increased uptake of fluorescently labeled *S*.*aureus* (i.e., 32 and 64μg/ml of βA1-42 increased uptake to 171±23 and 165±24% of control; p<0.03 for either one; n = 7). Since the initial experiments shown in Fig 7A appeared to show a continued rise at the highest concentration of peptides used we tested the remaining peptides at higher concentrations as shown in Fig 7B. In these experiments βA22-42 caused a marked increase in uptake of *E*.*coli* which greatly exceeded the effect seen with βA22-40. No significant effects were seen with either βA1-34, βA1-28 or βA33-40 (Fig 7A and 7B). However, some increase in E.coli uptake was seen with 32μg/ml of βA35-42 (Fig 7A).

## Discussion

**Fig 8:**
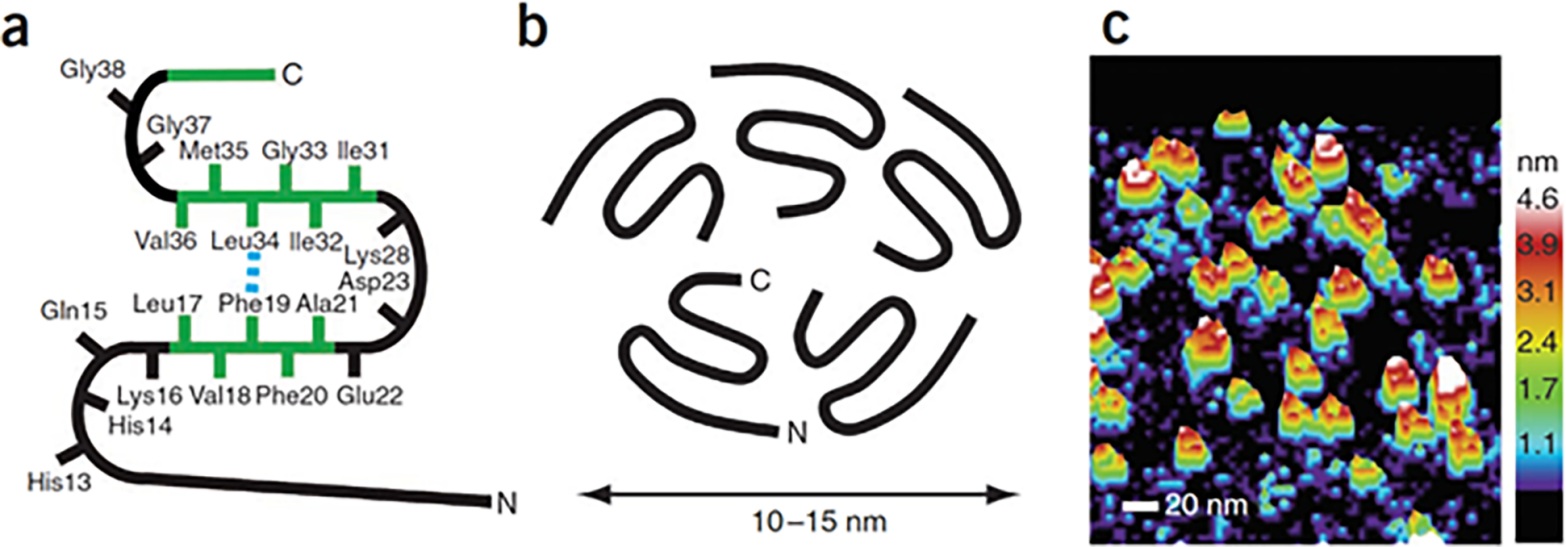
Mechanism through which C-terminal peptides contribute to formation of BA oligomers. This Fig was borrowed with permission from Ahmed et al [4]. Panel A shows a schematic of the structure of βA1-42 including the two intramolecular bonds present in the molecule. Panel B shows how the C-terminal loop formed by the bond between Ala42 and Met35 is involved in self aggregation of the peptide. Panel C shows atomic force microscopic images of oligomers formed by βA1-42.

These studies confirm the importance of the N-terminal amino acids Ile41 and Ala42 in the antiviral and antibacterial actions of βA. This was demonstrated by comparing activities of various peptides which contain (or did not contain) these amino acids. These residues are structurally important since they allow βA peptides to form the C-terminal loop between Met35 and Ala42 and this loop has been found to be critical in mediating oligomerization of βA peptides [4, 24]. Fig 8 was obtained by permission from Ahmed et al [4]. This Fig demonstrates how the loop formed by the C-terminal amino acids allows alignment of this end of the molecule in a central cavity of the oligomers. The ability of βA35-42 to induce aggregation of IAV indicates a relationship between viral aggregation and oligomerization. We have obtained similar findings with oligomerized forms of defensins or surfactant protein D and aggregation of IAV [25, 26].

βA1-42 is more neurotoxic than βA1-40 and its concentration is selectively increased in amyloid fibrils in the plaques of Alzheimer’s disease patients [1]. The greater neurotoxicity of βA1-42 as compared to βA1-40 is felt to relate to the greater propensity of the full length peptide to form neurotoxic oligomers. Analysis of small fragments of amino acids corresponding to βA 35–40 (MVGGVV), βA37-42 (GGVVIA), and βA35-42 (MVGGVVIA) by Wagoner et al using simulation kinetics and energetics revealed the increased propensity of βA35-42 assembled into unorganized oligomers as well [24]. The findings indicated that this N-terminal domain of protein is involved in self-aggregation. This self-aggregating property also appears to allow for aggregation of pathogens. Overall our findings and recent findings of Kumar and Spitzer [12, 13] suggest the hypothesis that the very property that makes βA1-42 neurotoxic also mediate antiviral and antibacterial or antifungal activities.

Our results also indicate that the two C-terminal amino acids of βA1-42 contribute to its ability to modulate pathogen interactions with neutrophils. βA22-42 had particularly potent abilities to increase neutrophil uptake of IAV or *E*.*coli*, exceeding that of the full length peptide. In addition, the various N-terminal fragments of βA that did not include the C-terminus lacked the ability to increase viral uptake or neutrophil respiratory burst responses. βA35-42 also increased slightly neutrophil uptake of IAV and bacteria, while βA33-40 did not. βA1-42 contains another turn formed by a salt bridge between Lys28 and Asp23 forming a β-turn-β conformation in the full length peptide (see Fig 8A). This salt bridge would be retained in βA22-42 but not in βA35-42. This may explain the enhanced antiviral activity of βA22-42 compared to βA35-42. βA1-42 has been reported to bind to some innate immune receptors on phagocytes (e.g. formyl peptide receptor 2, TLR2 and RAGE) [16, 27] and future studies could determine if the shortened fragments of βA retain the ability to bind these receptors.

## Supporting information

S1 Fig LDH assays on MDCK cells treated with βA preparations—LDH assays (n = 4). MDCK cells were treated with the indicated βA preparations at 50 μg/ml for 45 minutes. At 37°C as in the infectious focus assay. LDH was measured by ELISA assay at 18 hrs following the manufacturer’s instruction (Clontech, Mountain View, CA).
(PPTX)

## References

[1] Dahlgren KN, Manelli AM, Stine WB Jr., Baker LK, Krafft GA, LaDu MJ. Oligomeric and fibrillar species of amyloid-beta peptides differentially affect neuronal viability. J Biol Chem. 2002;277(35):32046–53. doi: 10.1074/jbc.M201750200 . 12058030 10.1074/jbc.M201750200

[2] Masters CL, Selkoe DJ. Biochemistry of amyloid beta-protein and amyloid deposits in Alzheimer disease. Cold Spring Harbor perspectives in medicine. 2012;2(6):a006262 doi: 10.1101/cshperspect.a006262 ; PubMed Central PMCID: PMC3367542. 22675658 10.1101/cshperspect.a006262 PMC3367542

[3] Selkoe DJ. Biochemistry and molecular biology of amyloid beta-protein and the mechanism of Alzheimer's disease. Handbook of clinical neurology. 2008;89:245–60. doi: 10.1016/S0072-9752(07)01223-7 . 18631749 10.1016/S0072-9752(07)01223-7

[4] Ahmed M, Davis J, Aucoin D, Sato T, Ahuja S, Aimoto S, et al Structural conversion of neurotoxic amyloid-beta(1–42) oligomers to fibrils. Nat Struct Mol Biol. 2010;17(5):561–7. doi: 10.1038/nsmb.1799 ; PubMed Central PMCID: PMC2922021. 20383142 10.1038/nsmb.1799 PMC2922021

[5] Kandel R, White M, Hartshorn K. Is Alzheimer associated beta amyloid an innate immune protein? 2016 In: Exploring New Findings on Amyloidosis [Internet]. http://www.intechopen.com/books: In-Tech Open Books.

[6] Zhang J, Liu J, Katafiasz B, Fox H, Xiong H. HIV-1 gp120-induced axonal injury detected by accumulation of beta-amyloid precursor protein in adult rat corpus callosum. J Neuroimmune Pharmacol. 2011;6(4):650–7. doi: 10.1007/s11481-011-9259-6 . 21286834 10.1007/s11481-011-9259-6 PMC3165079

[7] Wozniak MA, Itzhaki RF, Shipley SJ, Dobson CB. Herpes simplex virus infection causes cellular beta-amyloid accumulation and secretase upregulation. Neurosci Lett. 2007;429(2–3):95–100. doi: 10.1016/j.neulet.2007.09.077 . 17980964 10.1016/j.neulet.2007.09.077

[8] Jang H, Ma B, Lal R, Nussinov R. Models of toxic beta-sheet channels of protegrin-1 suggest a common subunit organization motif shared with toxic alzheimer beta-amyloid ion channels. Biophys J. 2008;95(10):4631–42. doi: 10.1529/biophysj.108.134551 . 18708452 10.1529/biophysj.108.134551 PMC2576390

[9] Papareddy P, Morgelin M, Walse B, Schmidtchen A, Malmsten M. Antimicrobial activity of peptides derived from human ss-amyloid precursor protein. J Pept Sci. 2012;18(3):183–91. doi: 10.1002/psc.1439 . 22249992 10.1002/psc.1439

[10] Soscia SJ, Kirby JE, Washicosky KJ, Tucker SM, Ingelsson M, Hyman B, et al The Alzheimer's disease-associated amyloid beta-protein is an antimicrobial peptide. PLoS One. 2010;5(3):e9505 doi: 10.1371/journal.pone.0009505 . 20209079 10.1371/journal.pone.0009505 PMC2831066

[11] Bourgade K, Garneau H, Giroux G, Le Page AY, Bocti C, Dupuis G, et al beta-Amyloid peptides display protective activity against the human Alzheimer's disease-associated herpes simplex virus-1. Biogerontology. 2015;16(1):85–98. doi: 10.1007/s10522-014-9538-8 . 25376108 10.1007/s10522-014-9538-8

[12] Kumar D, Choi S, Washicosky K, Eimer W, Tucker S, Ghofrani J, et al Amyloid-b peptide protects against microbial infection in mouse and worm models of Alzheimer’s disease. Science Translational Medicine. 2016;8:340ra72 doi: 10.1126/scitranslmed.aaf1059 27225182 10.1126/scitranslmed.aaf1059 PMC5505565

[13] Spitzer P, Condic M, Herrmann M, Oberstein TJ, Scharin-Mehlmann M, Gilbert DF, et al Amyloidogenic amyloid-beta-peptide variants induce microbial agglutination and exert antimicrobial activity. Scientific reports. 2016;6:32228 doi: 10.1038/srep32228 ; PubMed Central PMCID: PMCPMC5021948. 27624303 10.1038/srep32228 PMC5021948

[14] Oppenheim JJ, Yang D. Alarmins: chemotactic activators of immune responses. Curr Opin Immunol. 2005;17(4):359–65. doi: 10.1016/j.coi.2005.06.002 . 15955682 10.1016/j.coi.2005.06.002

[15] Jana M, Palencia CA, Pahan K. Fibrillar amyloid-beta peptides activate microglia via TLR2: implications for Alzheimer's disease. J Immunol. 2008;181(10):7254–62. . 18981147 10.4049/jimmunol.181.10.7254 PMC2701549

[16] Richard KL, Filali M, Prefontaine P, Rivest S. Toll-like receptor 2 acts as a natural innate immune receptor to clear amyloid beta 1–42 and delay the cognitive decline in a mouse model of Alzheimer's disease. J Neurosci. 2008;28(22):5784–93. doi: 10.1523/JNEUROSCI.1146-08.2008 . 18509040 10.1523/JNEUROSCI.1146-08.2008 PMC6670789

[17] White MR, Kandel R, Tripathi S, Condon D, Qi L, Taubenberger J, et al Alzheimer's associated beta-amyloid protein inhibits influenza A virus and modulates viral interactions with phagocytes. PLoS One. 2014;9(7):e101364 doi: 10.1371/journal.pone.0101364 ; PubMed Central PMCID: PMC4079246. 24988208 10.1371/journal.pone.0101364 PMC4079246

[18] Hartshorn KL, Collamer M, Auerbach M, Myers JB, Pavlotsky N, Tauber AI. Effects of influenza A virus on human neutrophil calcium metabolism. J Immunol. 1988;141(4):1295–301. 3135328

[19] Doss M, Ruchala P, Tecle T, Gantz D, Verma A, Hartshorn A, et al Hapivirins and diprovirins: novel theta-defensin analogs with potent activity against influenza A virus. J Immunol. 2012;188(6):2759–68. doi: 10.4049/jimmunol.1101335 . 22345650 10.4049/jimmunol.1101335 PMC3294087

[20] Hartshorn K, Crouch E, White M, Colamussi M, Kakkanatt A, Tauber B, et al Pulmonary surfactant proteins A and D enhance neutrophil uptake of bacteria. Amer J Physiol. 1998;274:L958–69. 9609735 10.1152/ajplung.1998.274.6.L958

[21] Hartshorn KL, Wright J, Collamer MA, White MR, Tauber AI. Human neutrophil stimulation by influenza virus: relationship of cytoplasmic pH changes to cell activation. Am J Physiol. 1990;258(6 Pt 1):C1070–6. doi: 10.1152/ajpcell.1990.258.6.C1070 . 2113768 10.1152/ajpcell.1990.258.6.C1070

[22] Tecle T, White MR, Gantz D, Crouch EC, Hartshorn KL. Human neutrophil defensins increase neutrophil uptake of influenza A virus and bacteria and modify virus-induced respiratory burst responses. J Immunol. 2007;178(12):8046–52. . 17548642 10.4049/jimmunol.178.12.8046

[23] Daigneault DE, Hartshorn KL, Liou LS, Abbruzzi GM, White MR, Oh SK, et al Influenza A virus binding to human neutrophils and cross-linking requirements for activation. Blood. 1992;80(12):3227–34. . 1334733

[24] Wagoner VA, Cheon M, Chang I, Hall CK. Impact of sequence on the molecular assembly of short amyloid peptides. Proteins. 2014;82(7):1469–83. doi: 10.1002/prot.24515 ; PubMed Central PMCID: PMC4217531. 24449257 10.1002/prot.24515 PMC4217531

[25] White M, Kingma P, Tecle T, Kacak N, Linders B, Heuser J, et al Multimerization of surfactant protein D, but not its collagen domain, is required for antiviral and opsonic activities related to influenza virus. J Immunol. 2008;181(11):7936–43. . 19017984 10.4049/jimmunol.181.11.7936

[26] Doss M, White MR, Tecle T, Gantz D, Crouch EC, Jung G, et al Interactions of alpha-, beta-, and theta-defensins with influenza A virus and surfactant protein D. J Immunol. 2009;182(12):7878–87. doi: 10.4049/jimmunol.0804049 . 19494312 10.4049/jimmunol.0804049

[27] Slowik A, Merres J, Elfgen A, Jansen S, Mohr F, Wruck CJ, et al Involvement of formyl peptide receptors in receptor for advanced glycation end products (RAGE)—and amyloid beta 1-42-induced signal transduction in glial cells. Mol Neurodegener. 2012;7:55 doi: 10.1186/1750-1326-7-55 . 23164356 10.1186/1750-1326-7-55 PMC3519738

