# Supplementary material for: Critical role of C-terminal residues of the Alzheimer's associated β-amyloid protein in mediating antiviral activity and modulating viral and bacterial interactions with neutrophils
Source: PLoS One. 2018 Mar 16;13(3):e0194001. doi: 10.1371/journal.pone.0194001 (PMC5856391; doi:10.1371/journal.pone.0194001)
Supplement: S1 Fig — MDCK cells were treated with the indicated βA preparations at 50 μg/ml for 45 minutes. At 37°C as in the infectious focus assay. LDH was measured by ELISA assay at 18 hrs following the manufacturer’s instruction (Clontech, Mountain View, CA). (PPTX) [file pone.0194001.s001.pptx]

## Slide 1
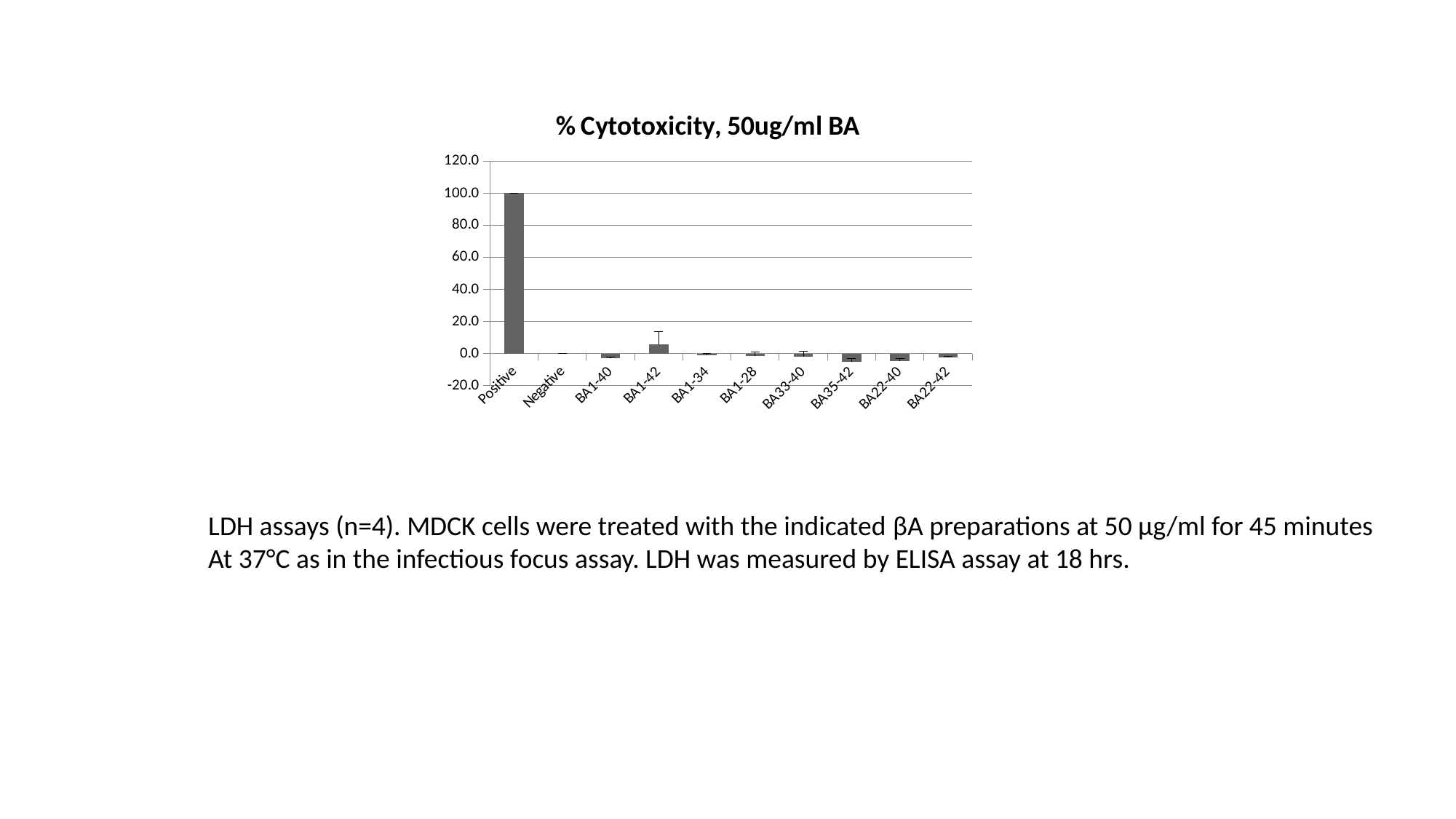

### Chart: % Cytotoxicity, 50ug/ml BA
| Category | % Cytotoxicity |
|---|---|
| Positive | 100.0 |
| Negative | 0.0 |
| BA1-40 | -3.0093260162918245 |
| BA1-42 | 5.536512852186668 |
| BA1-34 | -1.0225702912156416 |
| BA1-28 | -1.5852145889283016 |
| BA33-40 | -1.893729122070685 |
| BA35-42 | -5.127805437763028 |
| BA22-40 | -4.82346528025656 |
| BA22-42 | -2.5358008243679464 |LDH assays (n=4). MDCK cells were treated with the indicated βA preparations at 50 µg/ml for 45 minutes
At 37°C as in the infectious focus assay. LDH was measured by ELISA assay at 18 hrs.
